# Mass Transport through Nanostructured Membranes: Towards a Predictive Tool

**DOI:** 10.3390/membranes6040049

**Published:** 2016-12-02

**Authors:** Siavash Darvishmanesh, Bart Van der Bruggen

**Affiliations:** ProcESS—Process Engineering for Sustainable Systems, Department of Chemical Engineering, KU Leuven, Celestijnenlaan 200F, Leuven B-3001, Belgium; siavashd@princeton.edu

**Keywords:** solvent resistant nanofiltration, modelling, ceramic membranes

## Abstract

This study proposes a new mechanism to understand the transport of solvents through nanostructured membranes from a fundamental point of view. The findings are used to develop readily applicable mathematical models to predict solvent fluxes and solute rejections through solvent resistant membranes used for nanofiltration. The new model was developed based on a pore-flow type of transport. New parameters found to be of fundamental importance were introduced to the equation, i.e., the affinity of the solute and the solvent for the membrane expressed as the hydrogen-bonding contribution of the solubility parameter for the solute, solvent and membrane. A graphical map was constructed to predict the solute rejection based on the hydrogen-bonding contribution of the solubility parameter. The model was evaluated with performance data from the literature. Both the solvent flux and the solute rejection calculated with the new approach were similar to values reported in the literature.

## 1. Introduction

Solvent-resistant nanofiltration (SRNF) has become the focus of scientific attention as an alternative molecular purification technology for solutes [1,2,3]. SRNF can be used to separate small molecules in the molar mass range of 200 to 2000 g·mol^−1^ from organic solvents [4] In addition to the development of new SRNF membranes, understanding the fundamentals of transport of solvents and solutes through the membrane, and model-wise translation of first principles, is essential. A comprehensive transport model, which takes into account properties of the membrane, solvent and solutes, should allow for a correct prediction of the separation performance, which is necessary for general industrial applications of this separation technology. The literature on transport mechanisms in SRNF shows that mutual interactions between the solvents and the polymer at the membrane top layer (i.e., the separating layer) determine the separation performance [5]. The solvent that sorbs preferentially into the membrane surface is the main component at the permeate side of the membrane. A large influence from the interactions on the separation of solutes was also reported in the literature [6,7]. The affinity between the solvent and the polymeric membrane and between the solvent and solute determine the rejection. When the affinity between solute and membrane is higher than between solute and solvent, a lower rejection is expected [8].

One of the main challenges reported in the literature regarding the derivation of a transport model for SRNF is the swelling of the membrane structure due to the interaction of organic solvents with polymeric membranes [9,10]. Due to swelling the membrane structure changes, which affects the membrane performance. However, after crosslinking the membrane’s top layer and improving the membrane stability in organic solvents, membrane swelling becomes less of an issue [11,12].

The performance of SRNF as a membrane process is determined by the solvent permeability, the solute rejection and the recovery of the membrane unit. Various research groups have studied the fundamentals of transport phenomena in SRNF. Most transport studies for SRNF are restricted to experimental measurement and verification of pure solvent permeability through SRNF membranes [13,14,15,16,17,18,19,20,21,22,23,24]. At the same time, some studies also examine the rejection performance of SRNF [7,9,10,25,26,27,28,29,30,31,32,33]. The literature confirms that the membrane-solvent complex interactions play a determining role in the prediction of solvent fluxes through SRNF membranes [13,14,18,19,28,34,35,36,37,38]. Consequently, the information presented about the separation performance of one membrane-solvent system is not straightforwardly applicable to another system. In organic solvent systems, the molecular weight cut-off (MWCO) appears in fact to be solvent-dependent and cannot be used further for the estimation of solute rejection in SRNF membranes. This fact is proven by observation of solute rejection in organic solvents. Lower rejections with a commercial polydimethylsiloxane (PDMS)-made membrane for vitamin B12, brilliant blue R and safranin O in methanol than could be expected based on the specified MWCO were found by Whu et al. [25] and Yang et al. [15]. White et al. [16] suggested that the pore structure of the membranes and polymer–solvent interactions have a significant effect on the performance of the membrane. Interaction between the solvent and the membrane is not the only factor, as solute–polymer and solvent–solute interactions also determine the rejection performance. For a system with a stronger affinity between solute and membrane than between solute and solvent or between solvent and membrane, a lower rejection would be expected [7]. The understanding of the transport phenomena in SRNF is also inspired by an explanation of these interactions. SRNF membranes are on the range of micro-porous to dense membranes, and the discussion whether solute transport occurs by convection or diffusion is still ongoing. Bhanushali et al. [7] applied a systematic approach to get into the solute transport in SRNF. They found that solute–solvent coupling is a major factor in solute transport through membranes. Therefore, mutually convective and diffusive contributions of transport should be applied in describing solute migration through the membrane. In contrast, Silva et al. [20] applied simplified descriptions of both approaches (solution-diffusion approach as well as pore flow model) to their experimental results. The deviations of these models with experimental data were evaluated. They claimed that solution diffusion gives a better fitting value of a solution consisting of methanol and dimethyl succinate through a polyimide membrane in comparison with the pore flow model. White et al. [16] also claimed that solute transport through nanofiltration (NF) membranes occurs solely by diffusion and that therefore only a solution-diffusion approach should be considered. The permeation of various mixtures of hydrocarbons (pentane–decane and pentane–dodecane) through laboratory-made dense PDMS/PAN membranes has been measured by Dijkstra et al. [9]. Their experimental results have been modeled for two existing models, namely the solution-diffusion-with-imperfections model and the Maxwell–Stefan transport equation, which consider both diffusive and viscous flow. Applying the Maxwell–Stefan transport equation gave a more realistic outcome. Darvishmanesh et al. [28] proposed a model equation for the permeation of organic solvents from different chemical classes through polymeric membranes by improving the solution-diffusion with imperfection model by adding entrance resistance effects (surface tension, β), and the polarity effect between the membrane and the solvent (dielectric constant, α) within the original equation. Later on they introduced a new coupled series-parallel resistance model for transport of solvents through ceramic NF membranes. They proposed a semi-empirical correlation to describe the solvent flux, $J_{v}$, as governed by surface, pore and membrane resistances [39]. Robinson et al. [19] correlated the permeability of different paraffins including normal, branched and cyclic alkanes with viscous transport through a laboratory made dense PDMS membrane using the Hagen–Poiseuille model (linearity between solvent flux ($J_{v}$) and $\frac{\Delta P}{\mu }$).

(1) $$J_{v}=\frac{\epsilon r_{p}{}^{2}}{8\eta \tau }\frac{\Delta P}{\Delta x}$$

However, for each group of hydrocarbons, they found a different gradient $\frac{\epsilon r_{p}{}^{2}}{8\Delta x\tau }$. They explained this phenomenon by the change of membrane porosity due to swelling of membrane structure. They also showed that a relationship exists between the calculated gradient and the Hildebrand solubility parameter. Similar solubility parameters found between solvent and the membrane could result in swelling of the membrane matrix and open the so-called channel leading to pore-flow type behavior. It is, however, clear that a model in which the viscosity is the only solvent parameter is inadequate for the description of organic solvent fluxes. For instance, for hydrophilic membranes the water flux is higher than the n-Hexane flux through the same membrane, while the viscosity of the n-Hexane is about three times lower than water. Tsuru et al. [40,41] used silica–zirconia (Si–Zr) membranes in their study with different MWCO values. They reported that the solvent transport mechanism through (Si–Zr) membranes deviates from the viscous-flow mechanism due to reduction of the membrane’s pore size through solvent adsorption on membrane pore wall [42]. Later they claimed that alcohol permeation through hydrophobic modified (Si–Zr) membranes can be modeled by the use of Hagen–Poiseuille equation, corresponding to viscous-flow [43].

Clearly, no agreement on the exact mechanism for solute and solvent transport in SRNF has been reached yet. Identification of this transport mechanism is required to develop a general mathematical model for solute transport in SRNF, which would determine the efficiency of SRNF process. The literature on SRNF modeling shows that most transport models for solvent filtration through membranes are limited to specific experimental data and lack generalization due to the absence of a fundamental basis to describe the phenomena involved in solvent and solute transport through nanostructured membranes. In this study, a general model to describe the permeation of organic solvent and separation of solutes will be developed based on physico-chemical mechanisms involved in permeation. The model is based on the Hagen–Poiseuille pore flow mechanism. The influence of the permeating mixture on the sorption and the interaction with the membrane is taken into account. Finally, this new model will challenged with available data from the literature for SRNF.

## 2. Modeling Approach

**Figure 1:**
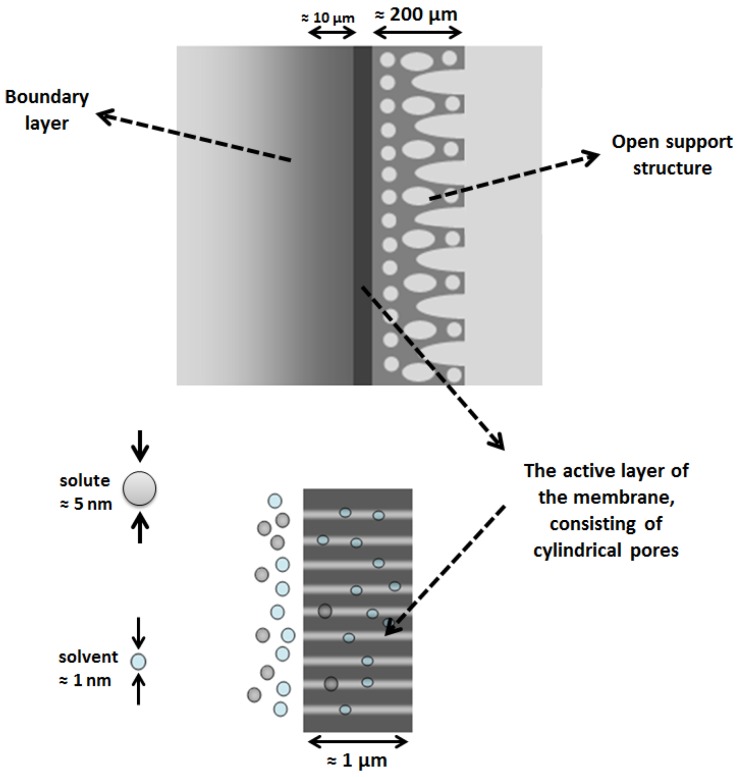
Schematic representation of SRNF membrane, dimension of parameters and membrane.

The nominal pore size (pore diameter) of NF membranes is typically about 1–2 nm. Most studies on modelling of solvent and solute transport through SRNF membranes consider the presence of pores with diameter of 1–2 nm in the membrane top-layer as bundles of capillary tubes. In such membranes, the Hagen–Poiseuille type equation can describe the relationship between the solvent flux and applied pressure. In this type of membrane, transport is mainly governed by the boundary layer near the membrane surface. Solutes will be more concentrated and accumulate near the surface. Concentration polarisation may affect significantly the solvent flux. Inside the membrane, transport occurs by viscous flow. The solutes always accumulate near the membrane, so that a turbulent flow is required to minimize the concentration polarisation effect. The active layer of the membrane (Figure 1) is typically around 1 µm thick. It consists of parallel cylindrical pores with a diameter of 1–2 nm, i.e., the same size as the solutes or smaller. These exclude the solutes. This active layer is so thin that it cannot be under high pressure on its own, so the membrane has a support layer that is much thicker. The mass transfer resistance of this layer is small, and could be neglected. The boundary layer thickness is around a few micrometers and the thickness of the entire membrane with the support layer is a few hundred micrometers.

### 2.1. Solvent Transport inside Pores

The viscosity of a solvent in narrow NF pores may not be the same as in the bulk solvent. The use of the bulk solvent viscosity may cause deviations when the flux of different groups of solvents is plotted versus the bulk viscosity. The solvent permeability may be decreased due to smaller pore size caused by orientation of the solvent molecules at the pore wall. This effect depends on interaction of solvent and membrane due to the hydrophobicity and hydrophilicity of the membrane. Dias et al. [44] studied the structure of water inside the NF/RO membranes and concluded that for a hydrophobic membrane, weakly H-bounded water clusters form, which has a strong influence on the permeation performance. It is known that the viscosity will increase with decreasing pore radius; however, this is difficult to calculate due to the small NF pore sizes. Bowen et al. [45] suggested that the presence of one layer of adsorbed water molecules (*d* = 0.28 nm) at the pore wall increases the viscosity around 10 times. They suggested the average pore viscosity to be:

(2) $$\frac{\eta_{pore}}{\eta_{solvent}}=1+18\left(\frac{d_{solvent}}{r_{p}}\right)-9{\left(\frac{d_{solvent}}{r_{p}}\right)}^{2}$$

**Figure 2:**
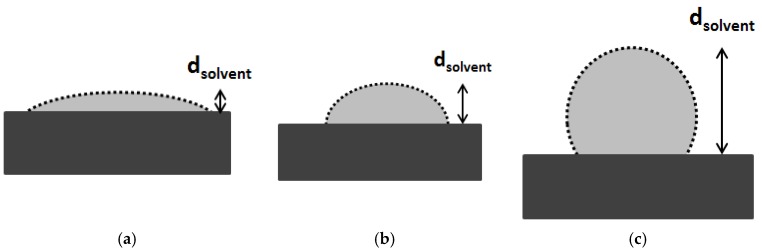
Schematic representation of a solvent drop on membrane surface with different degree of hydrophobicity. (**a**) Hydrophilic membrane and polar solvent or Hydrophobic membrane and apolar solvent; (**b**) Semi-Hydrophilic membrane and polar solvent and (**c**) Hydrophilic membrane and apolar solvent or Hydrophobic membrane and polar solvent.

Unfortunately, Bowen et al. [45] did not discuss the effect of membrane hydrophobicity on the viscosity and the formation of the water layer. It is clear that the adhesive forces between a solvent and membrane cause a liquid drop to spread across the membrane pore wall (Figure 2a). Cohesive forces within the solvent cause the drop to rise up and avoid contact with the surface (Figure 2b). Due to this effect the solvent layer thickness inside the pore may differ significantly.

In this study, a new parameter was introduced into Equation (2) to correct the solvent layer size on membrane pore walls:

(3) $$\frac{\eta_{pore}}{\eta_{solvent}}=1+18\left(\frac{\psi d_{solvent}}{r_{p}}\right)-9{\left(\frac{\psi d_{solvent}}{r_{p}}\right)}^{2}$$

ψ is the solvent layer size correction factor, and its value state between 0 and 1. 0 is the value related to the perfect wetting or strong interaction between membrane pore walls, and the solvent and 1 is for very weak interaction or low wettability or a non-wettable surface. In this way for a hydrophobic NF membrane, the ‘internal’ viscosity of water increases greatly and as a consequence, the flux drops. However, for an apolar solvent such as n-Hexane, the viscosity does not change for a hydrophobic membrane, and the flux remains high. Pore viscosity will be substituted with solvent viscosity in Equation (1), and solvent flux can be calculated in this way. ψ should be fitted for each solvent separately to get the best fit for the solvent permeability through the membrane.

The hydrophobic effect shows the affinity of polar molecules to exclude non-polar molecules, which leads to separation and segregation of polar and non-polar substances. Highly active hydrogen bonds between molecules of polar compounds, such as hydroxyl groups in water and methanol, cause a hydrophobic effect and provoke a low contact angle with a hydrophilic membrane. However, a hydrocarbon molecule, for example n-Hexane, is unable to form hydrogen bonds with hydrophilic substrate and yields a high contact angle with the hydrophilic membrane. In this way, the hydrogen-bonding contribution of the material could also indicate the degree of their hydrophobicity/hydrophilicity towards each other. The hydrogen-bonding affinity of solvent and membrane can be attributed to ψ, the solvent layer size correction factor, indicated in Equation (2). When the solvent and the membrane have a high affinity for strong hydrogen bonding, the value tends to be 0 (Water-TiO_2_) and one for a system with no affinity (n-Hexane-TiO_2_). Between these extremes the solvent layer thickness was found to change with the affinity in a logarithmic way. In order to enhance the user friendliness of the model this logarithmic trend was discretized corresponding to four different solvent categories. Table 1, shows the suggested values for the correction factor of different filtration systems which were obtained through fitting with available experimental data. For the system with very high affinity like cellulose acetate and water, this value is 0.001. However, for the system of no affinity (cellulose acetate and n-Hexane), ψ is 1.

**Figure 3:**
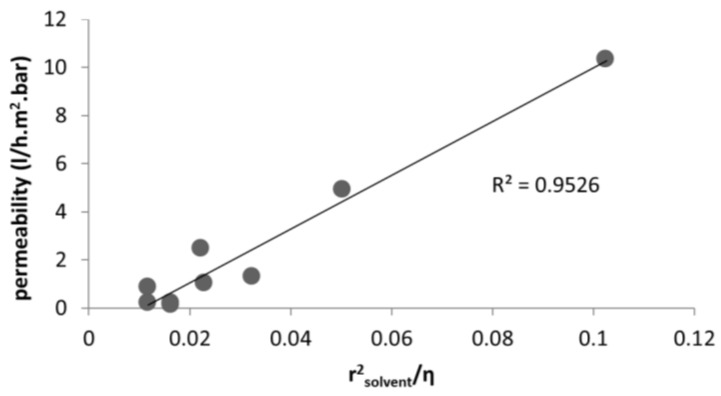
Solvent permeability versus $r_{\mathrm{solvent}}^{2}/\eta$ studied by [46].

**Figure 4:**
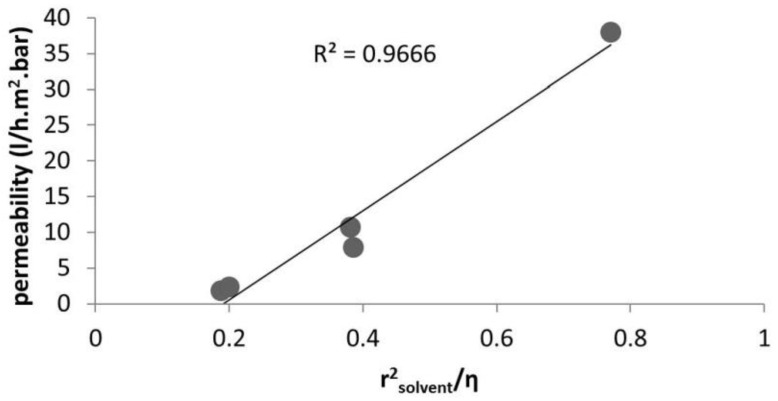
Solvent permeability versus $r_{\mathrm{solvent}}^{2}/\eta$ studied by [40].

**Figure 5:**
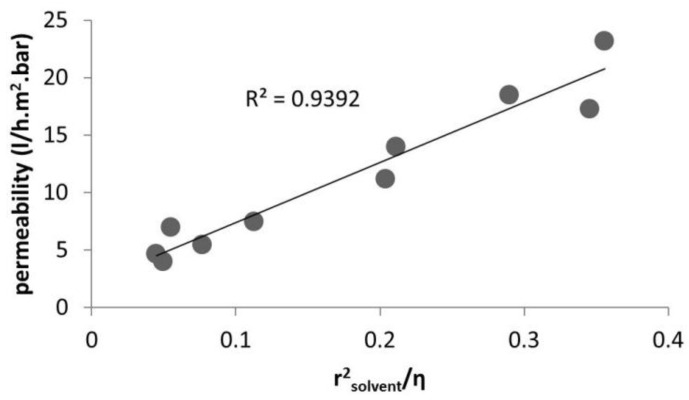
Solvent permeability versus $r_{\mathrm{solvent}}^{2}/\eta$ studied by [40].

Table 2, Table 3 and Table 4 summarize the solvent permeability across a ceramic membrane reported by three different research groups in the literature. The reason to challenge our model with these data is the availability of the pore size of the membrane as well as elimination of the swelling effect. It should be noted that the proposed model could be applied to a new generation of polymeric membranes (for example DuraMem^®^), if the pore size were available. Unfortunately, for all studies [40,46] no data were found regarding the porosity of the membrane, so that the results have to be plotted in different figures (Figure 3, Figure 4 and Figure 5). In this way we consider a similar porosity of the membrane in each separate study.

The solvent molecular diameters were calculated using chemistry software for molecular modeling (Hyperchem) [47]. This software determines an effective diameter by taking into account three parameters: the molecular structure, the chemical bond length and the bonding angles of the molecules. Dobrak et al. [48] demonstrated the procedure to use the software to calculate the effective solvent molecular diameters.

As can be seen in Figure 3 and Figure 4, the pore flux mechanism with the corrected viscosity can successfully predict the solvent flux through the membrane. The fitting R-square is improved by applying the different porosity value for each specific membrane. Table 4 present the solvent permeability through a ceramic NF membrane for a wide range of solvents (polar and non-polar). Figure 5 plots the permeability versus *r*^2^_solvent_/*η*. As is evident, by applying the viscous correction factor and correcting the solvent layer thickness inside pores to estimate real solvent viscosity inside pores, the solvent permeability through the NF membrane follows a pore-flow type of equation (flux depends on viscosity).

### 2.2. Solute Transport inside Pores

It is known from the literature that solute transport is influenced by solute–solvent–membrane interactions [6,8,14,36]. Several research groups tried to understand the mechanism of solute rejection in SRNF. The effect of solubility, dipole moment, surface energy, molecular size as well as dielectric constant are the most discussed parameters in literature. However, the results obtained by each specific system of study cannot be applied to a new system due to the lack of generalisation.

**Figure 6:**
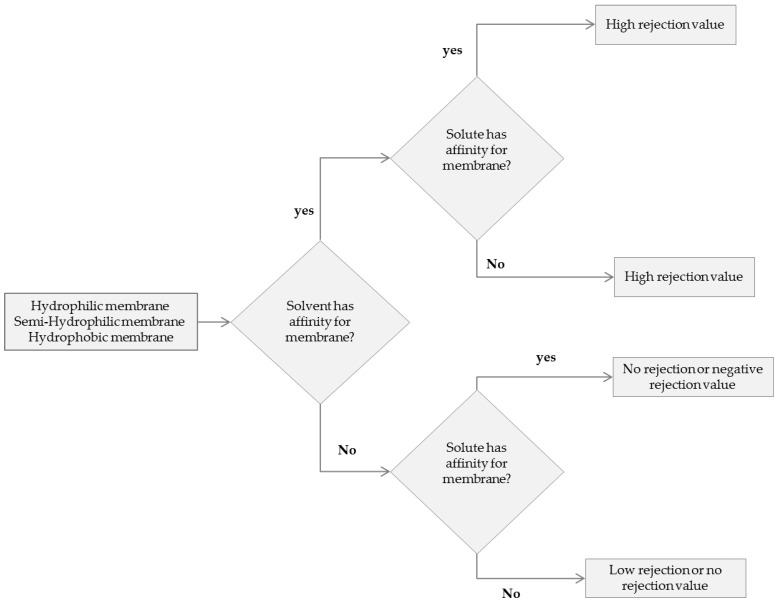
Scheme to predict the expected rejection of solutes with a given membrane. Solute and solvent affinity can be evaluated by the hydrogen-bonding ability of the membrane.

One of the parameters not mentioned and discussed clearly in literature is the hydrogen bonding capability of the solvent–solute–membrane system and its influence on the SRNF performance. Hydrogen bonds are much weaker than normal covalent or ionic chemical bonds but still represent very important electrostatic attractions. Hydrogen bonds are formed between the slightly positive parts of an individual solvent molecule and slightly negative parts of an adjacent molecule (or vice versa). This parameter can be related to the hydrogen-bonding contribution of Hansen solubility parameter ($\delta_{h}$) for solvent, solute and membranes. In a system with high capability of hydrogen bonding between solvent and membrane (a hydrophilic membrane and a polar solvent), a high flux is to be expected. For a hydrophilic membrane as solvent, $\delta_{h}$ decreases the flux decrease as well. For hydrophilic membrane made of, e.g., cellulose acetate or TiO_2_, a high rejection is observed for polar solvents such as water and alcohol [49,50]. Due to the high hydrogen bonding capability of water and methanol with TiO_2_, and also their low viscosity a higher flux is observed. Higher fluxes result in a high rejection. Koops et al. [50] studied the solute rejection and the solvent flux of linear hydrocarbons (*M*_w_ = 226–563 g/mol) and linear carboxylic acids (*M*_w_ = 228–340 g/mol) in ethanol and hexane as a function of the molar mass, the feed concentration and the transmembrane pressure. Ethanol has a higher affinity toward cellulose acetate compared to n-Hexane, resulting in a higher flux for ethanol, and a higher rejection of carboxylic acid and linear hydrocarbon compounds in ethanol, while for the same membrane, a negative rejection can be observed for carboxylic acid compound dissolved in n-Hexane due to the hydrogen bonding capability of carboxylic acid with the membrane over the n-Hexane ($\delta_{h}=0$) [50]. In the case of a semi-hydrophilic membrane, the solvent with hydrogen bonding capability closest to the membrane should have the highest flux. In this case a higher flux for the membrane is achieved for a solvent with the $\delta_{h}$ value close to the membrane and with the lowest viscosity. A solvent with high hydrogen bonding capability (like water) does not yield a higher flux. A higher flux of methanol over water has been observed for NF30 and NF-PES-10 (polyethersulfone membrane;$\;\delta_{h}=7.6\;{\mathrm{MPa}}^{\frac{1}{2}}$), due to higher affinity of water molecules towards other water molecules instead of the membrane [51]. For a hydrophobic membrane material such as polydimethylsiloxane (PDMS), the solvent with the lowest hydrogen bonding ability has the highest flux. Bhanushali et al. [7] present methanol, ethanol and hexane flux and azo dyes rejection over the membrane denoted as D Osmonics (a commercial PDMS based membrane). A low flux of alcohol and negative solute rejection has been observed for alcohols. The $\delta_{h}$ value of the PDMS membrane is around$\;0.3\;{\mathrm{MPa}}^{\frac{1}{2}}$, which indicates no affinity for hydrogen bonding. In this case the solvent with higher $\delta_{h}$ has the lowest flux. Negative rejection was the result of the higher affinity of azo dyes compared to methanol and ethanol towards the membrane. A lower flux of n-Hexane with negative rejection was also observed for a semi-hydrophilic polyimide membrane, due to the absence of hydrogen bonding of n-Hexane with the membrane matrix, and the higher affinity of dyes for the membrane. Figure 6 presents different phenomena that were observed for solute rejection.

As can be seen in Figure 6, the preferential interaction of solute and solvent towards the membrane is postulated to govern transport through the membrane.

The volume fractions of solute and solvent inside and outside the pores are not equal. An important reason for this is the effect of size exclusion. A nanostructured membrane excludes solutes larger than the pore size. However, due to the contribution of hydrogen bonding, the solute–membrane affinity is also thought to be important.

The size exclusion effect for cylindrical pores was given in the literature [52] as:

(4) $$K_{exclusion}={\left(1-\frac{d_{solute}}{d_{pore}^{\prime }}\right)}^{2}$$

**Figure 7:**
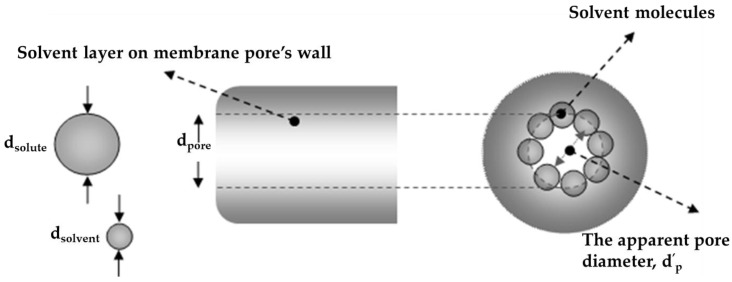
Schematic representation of SRNF membrane pores. The real pore size is smaller in contact with solvent.

This equation may apply for a membrane with very sharp MWCO, which indicates a unique pore size distribution along the membrane. Effect of solvent on nominal pore size of the membrane presented in Figure 7, is also considered in Equation (4). Literature study shows that solutes with larger diameter than pore size are not always rejected completely. This takes place due to the existence of pore sizes in the membrane surface larger than the pore size value reported by the manufacturer or the size measured experimentally. The occurrence of such pores has an irregular effect on the rejection of solutes. Due to the pore size distribution along the membrane surface, different rejection values can be considered depending on the solute size.

To include this effect on solute partitioning, the size exclusion effect can be rewritten as:

(5) $$K_{exclusion}={\left(1-\alpha \frac{d_{solute}}{d_{pore}^{\prime }}\right)}^{2}$$

α is a membrane property depending on the solute size and its value is between 0 and 1. Due to the pore size distribution of the membrane surface, different α values can be considered. The value of α can be fitted experimentally for each range of solute size independent from the solvent in use. However, for larger solute sizes, a larger α is expected. When the solute size is larger than the highest available pore size in the membrane surface, α value of 1 is to be assigned for those specific solutes.

The concentration inside the pores ($c_{inside}$) is related to the concentration outside the pores (on the surface;$\;c_{m}$) with the following equation:

(6) $$c_{inside}=K_{exclusion}\times c_{m}$$

**Figure 8:**
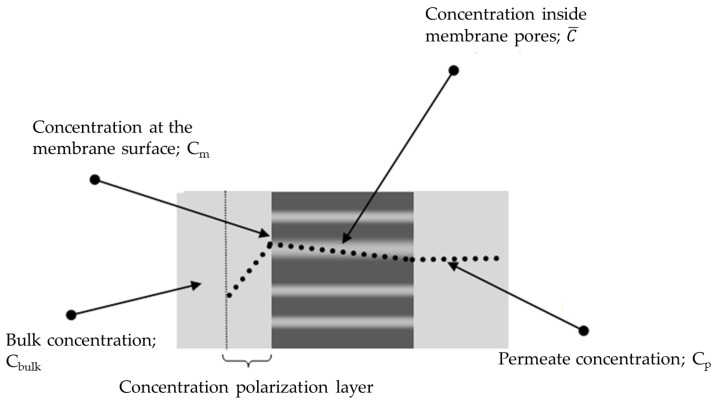
Concentration profile across the membrane.

The concentration profile through the membrane is presented in Figure 8. The total solute flux $J_{s}$ is related to the total volume flux, $J_{v}$ by means of the average concentration of solutes inside pores [52]: $J_{s}=\left(average\;concentration\;of\;solutes\;inside\;pores\right)\times J_{v}$ or

(7) $$J_{s}=\omega \frac{c_{inside}-c_{p}}{\bar{c}}J_{v}$$

**Figure 9:**
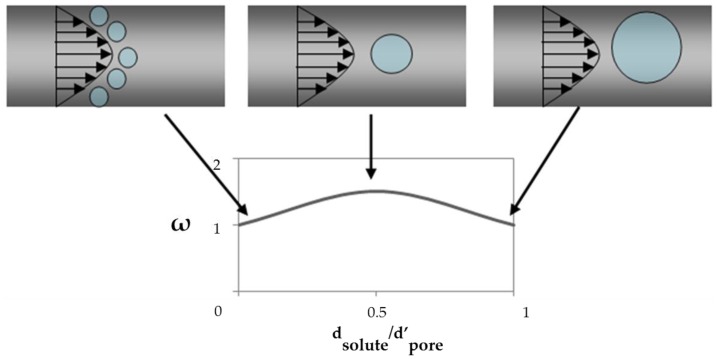
Solute velocity profile through the narrow pores, w is viscous correction factor.

ω is the viscous selectivity of solutes in pores. The solute tends to move into the center of pore. Thus, its velocity is a little higher than that of the solvent. In this way the viscous selectivity of solutes (ω) is larger than 1. Figure 9 shows this phenomenon. When the solute is much smaller than the pore, it moves with the same velocity as the solvent. This phenomenon also occurs when the solute just fits into the pore. The solute velocity has a maximum of about one and half times the water velocity [53].

ω can be calculated approximately with the following equation, proposed by Krishna et al. [53]

(8) $$\omega \approx 1+2\frac{d_{solute}}{d_{pore}^{\prime }}\left(1-\frac{d_{solute}}{d_{pore}^{\prime }}\right)$$

For $\frac{d_{solute}}{d_{pore}^{\prime }}>1$, ω can be considered to be 1. For sake of simplicity, we consider no change in the concentration profile inside the pores; consequently, the solute flux can be derived from the solvent flux by:

(9) $$J_{s}=\omega c_{inside}J_{v}$$

Equation (9) is further challenged with rejection values from other studies [48]. Table 5 presents the results for parameter used in Equations (5)–(9).

As can be seen in Table 5 the model fully predicts the rejection. The α value changes for each membrane and for each solute size. For larger solutes this value is higher (for brilliant blue, it is around two times higher than for bromothymol blue). As mentioned before, the α value is related to the membrane pore size distribution as well as solute size, which does not change with the solvent. Table 6 compares the other set of experimental rejection values with the proposed model. Since they used a similar type of membrane (TiO_2_ Fraunhofer IKTS, Dredsen, Germany), the α value should be the same. However, the cut-off value changes a bit from batch to batch (claimed by [49]), so α values change accordingly. The only difference was observed for the mixture of tridodecylamine in n-heptane. Since the hydrogen bonding contribution of the solubility parameter of n-heptane is 0, a higher amount of tridodecylamine can pass through the membrane due to the higher affinity of this solute toward the membrane compared to n-heptane.

It should be noted that the transport model suggested here can be applied to every nanostructured membrane with known pore size. It is also obvious that solvent filtration with a pore size in the range of 1–2 nm follows the pore flow transport mechanism. The only fitting parameter is α, which can be evaluated for different solute sizes based on the molecular weight or molecular size of solutes.

## 3. Conclusions

This manuscript presents predictive tools for the estimation of solvent and solute transport through SRNF membranes with known pore sizes. Solute, solvent and membrane parameters were identified, and the model presented in this work is based on the integration of different parameters that appear in existing transport models. The proposed model in this study could successfully predict the transport performance of SRNF ceramic membranes.

This newly presented model for solvent transport through SRNF-membranes shows that any transport model must contain a correction value to correct the viscosity inside the pores. Here, ψ was introduced to correct for the thickness of the solvent layer in membrane nano-pores based on the degree of hydrophobicity of the membrane and polarity of the solvent.

Another fitting parameter was introduced for the correction of the size exclusion effect during partitioning, which is caused by the membrane pore size distribution. The model was evaluated with a large database of experimental flux data from literature available for ceramic membranes with known pore sizes. Comparison between experimental and calculated rejection value shows that the proposed model could predict the rejection value satisfactorily. It can be concluded that the model presented here is suitable for membranes with pore sizes in NF range. This model may be applied for both hydrophilic and hydrophobic NF membranes.

## Figures and Tables

**Table 1:** The value of viscosity correction factor indicated in Equation (2).

High Affinity	Good Affinity	Moderate Affinity	No Affinity
0.001	0.01	0.1	1

**Table 2:** Solvent permeability through the ceramic membrane reported by [46] through ZrO_2_ and TiO_2_ membranes.

Membrane	Pore Radius of Membrane (nm)	Solvent	Solvent Molecule Diameter (nm)	Correction Factor *ψ*	*ψ* × Solvent Molecule Diameter (nm)	Permeability L/h·m^2^·bar
ZrO_2_	0.35	Ethanol	0.34	0.01	0.0034	10.3
		Heptane	0.57	1	0.57	2.5
		Toluene	0.5	1	0.5	1.3
	0.25	Ethanol	0.34	0.01	0.0034	4.9
		Heptane	0.57	1	0.57	0.9
		Toluene	0.5	1	0.5	0.2
TiO_2_	0.25	Ethanol	0.34	0.1	0.034	1
		Heptane	0.57	1	0.57	0.2
		toluene	0.5	1	0.5	<0.1

**Table 3:** Solvent permeability through the ceramic Si–Zr membrane reported by [40].

Pore Radius of Membrane (nm)	Solvent	Solvent Molecule Diameter (nm)	Correction Factor *ψ*	*ψ* × Solvent Molecule Diameter (nm)	Permeability L/h·m^2^·bar
0.6	methanol	0.27	0.1	0.02	7.9
	ethanol	0.34	0.1	0.0225	1.8
0.8	methanol	0.27	0.1	0.02	38
	ethanol	0.34	0.1	0.0225	10.7
	iso-propanol	0.39	0.1	0.0255	2.27

**Table 4:** Solvent permeability through the ceramic membrane reported by [49]. Pore radius of membrane (0.45 nm) through TiO_2_ manufactured by Fraunhofer IKTS (Dredsen, Germany).

Solvent	Solvent Molecule Diameter (nm)	Correction Factor *ψ*	*ψ* × Solvent Molecule Diameter (nm)	Permeability L/h·m^2^·bar
Methanol	0.27	0.01	0.0027	23.2
Ethanol	0.34	0.1	0.034	7.5
Iso-propanol	0.39	0.1	0.039	7
Ethyl actetate	0.71	0.1	0.071	11.2
THF	0.59	1	0.59	4
Heptane	0.57	1	0.57	5.5
Dichloromethane	0.36	0.1	0.036	11.1
Acetonitrile	0.37	0.1	0.037	17.3
Toluene	0.5	1	0.5	4.7
Water	0.21	0.01	0.0021	23.2

**Table 5:** Evaluating the proposed model by solvent flux and solute rejection value through the ceramic membrane reported by [48].

Membrane	Average Pore Size	MWCO	Solute	Solute Diameter	Solvent Name	Solvent Molecule Diameter (nm)	*ψ* × Solvent Molecule Diameter (nm)	k	α	ω	Cin	Cout	Calculated Rejection Value	Experimental Rejection Value
TiO_2_	0.9	275	Brilliant blue	0.58	Ethanol	0.34	0.034	0.001	0.8	1.00	3	0.00	0.999	0.991
TiO_2_	0.9	275	Bromothymol blue	0.49	Ethanol	0.34	0.034	0.317	0.4	1.00	3	0.95	0.683	0.670
TiO_2_	0.9	275	Bromothymol blue	0.49	Toluene	0.50	0.500	0.023	0.4	1.00	3	0.07	0.977	0.993
TiO_2_	1.2	650	Brilliant blue	0.58	Ethanol	0.34	0.034	0.051	0.8	1.01	3	0.15	0.946	0.955
TiO_2_	1.2	650	Bromothymol blue	0.49	Ethanol	0.34	0.034	0.349	0.5	1.01	3	1.05	0.548	0.555
ZrO_2_	1.2	600	Brilliant blue	0.58	Ethanol	0.34	0.003	0.254	0.5	1.06	3	0.76	0.741	0.700
ZrO_2_	1.2	600	Bromothymol blue	0.49	Ethanol	0.34	0.003	0.625	0.25	1.06	3	1.88	0.205	0.165
ZrO_2_	1.2	600	Bromothymol blue	0.49	Toluene	0.50	0.500	0.588	0.25	1.26	3	1.76	0.339	0.360

**Table 6:** Evaluating the proposed model by solvent flux and solute rejection value through the ceramic membrane reported by [44]. The pore size diameter is 0.9. Tridodecylamine: van der Waals diameter was 1.08 nm.

Solvent Name	k	α	ω	C_in_	C_out_	Calculated Rejection Value	Experimental Rejection Value
methanol	0.58	0.2	1.000	3.00	1.73	0.42	0.38
ethanol	0.77	0.1	1.000	3.00	2.30	0.23	0.22
isopropanol	0.76	0.1	1.000	3.00	2.29	0.24	0.21
ethyl acetate	0.55	0.2	1.000	3.00	1.64	0.45	0.51
THF	0.23	0.15	1.000	3.00	0.68	0.77	0.78
heptane	0.45	0.1	1.000	3.00	1.36	0.55	0.38
toluene	0.21	0.2	1.000	3.00	0.63	0.79	0.80
methanol	0.58	0.2	1.000	3.00	1.73	0.42	0.38
